# Clinical Outcomes with and without Adherence to Evidence-Based Medicine Guidelines for Lumbar Degenerative Spondylolisthesis Fusion Patients

**DOI:** 10.3390/jcm12031200

**Published:** 2023-02-02

**Authors:** Glenn A. Gonzalez, Guilherme Porto, Kevin Hines, Daniel Franco, Thiago S. Montenegro, Aria Mahtabfar, Matthew O’Leary, Jingya Miao, Sara Thalheimer, Joshua E. Heller, Ashwini Sharan, James Harrop

**Affiliations:** 1 Department of Neurosurgery, Thomas Jefferson University and Jefferson Hospital for Neuroscience, Philadelphia, PA 19107, USA; 2 Department of Neurosurgery, Spectrum Health/Michigan State University, Grand Rapids, MI 49503, USA; 3 Department of Medicine, Drexel University College of Medicine, Philadelphia, PA 19104, USA

**Keywords:** degenerative spondylolisthesis, evidence-based medicine, lumbar fusion, ODI

## Abstract

Introduction: Degenerative lumbar spondylolisthesis (DS) patients are treated with instrumented fusion, following EBM guidelines, and typically have excellent clinical outcomes. However, not all lumbar fusion procedures adhere to EBM guidelines, typically due to a lack of prospective data. Objective: This retrospective study compared outcomes of DS lumbar fusion patients treated according to EBM guidelines (EBM concordant) to lumbar fused patients with procedures that did not have clear EBM literature that supported this treatment, the goal being to examine the value of present EBM to guide clinical care. Methods: A total of 125 DS patients were considered EBM concordant, while 21 patients were EBM discordant. Pre- and postsurgical ODI scores were collected. Clinical outcomes were stratified into substantial clinical benefit (SCB ΔODI >10 points), minimal clinical importance benefit (MCID ΔODI ≥ 5 points), no MCID (ΔODI < 5 points), and a group that showed no change or worsening ODI. Fisher’s exact and χ2 tests for categorical variables, Student’s *t*-test for continuous variables, and descriptive statistics were used. Statistical tests were computed at the 95% level of confidence. Results: Analysis of 125 degenerative spondylolisthesis patients was performed comparing preoperative and postoperative (6 months) ODI scores. ODI improved by 8 points in the EBM concordant group vs. 2.1 points in the EBM discordant group (*p* = 0.002). Compliance with EBM guidelines was associated with an odds ratio (OR) of 2.93 for achieving MCID ([CI]: 1.12–7.58, *p* = 0.027). Conclusions: Patients whose lumbar fusions met EBM criteria had better self-reported outcomes at six months than those who did not meet the requirements. A greater knowledge set is needed to help further support EBM-guided patient care.

## 1. Introduction

Degenerative spondylolisthesis (DS) is a condition characterized by the forward slipping of one vertebral body onto the one below, caused by the degeneration of the facet joints and looseness of the ligaments. [1]. Lumbar DS 29 is a major cause of spinal canal and foraminal stenosis and often creates clinical symptoms of low-back and leg pain [1]. This condition tends to occur after 50 years of age, with a higher incidence in women [2]. Management of DS has been debated as multiple surgical treatment modalities are currently employed [3]. Over the last several decades, applying evidence-based medicine (EBM) principles has helped mold treatment recommendations and patient management [4]. The use of evidence-based medicine (EBM) for fusion criteria in managing degenerative spondylolisthesis (DS) is essential for providing high-quality care to patients. EBM is a process that involves utilizing the most current and reliable scientific evidence to inform clinical decision-making, and it is considered the standard of care modern medicine [2]. Studies have shown that the use of EBM fusion criteria can improve patient-reported outcome measures (PROM), such as Oswestry Disability Index (ODI) scores [2,3]. These criteria are designed to ensure that patients receive the most appropriate treatment and postoperative care, thereby reducing the likelihood of complications and enhancing recovery [2,4].

The North American Spine Society (NASS) has provided recommendations to address clinical conditions related to diagnosing and treating degenerative lumbar spinal stenosis [5]. Furthermore, the AANS/CNS 2014 lumbar fusion guidelines for stenosis with spondylolisthesis support surgical decompression and fusion as an effective treatment option for symptomatic stenosis associated with DS. This attained a level B recommendation or suggested treatment [5,6]. Patients’ management should be tailored to improve the quality of life. In order to objectively measure patient outcomes, it is essential to have a baseline understanding of their health condition and to record changes. Therefore, the patient-reported outcome measure (PROM) was used, specifically the Oswestry Disability Index. This study aimed to compare patients who underwent lumbar fusion surgery for DS using the EBM fusion criteria and its effect on patient-reported outcome measures (PROM), specifically Oswestry Disability Index (ODI) scores, after a six-month follow-up.

## 2. Materials and Methods

### 2.1. Acquisition of Data

This was a single-center, observational, retrospective cohort study used to evaluate the use of EBM guidelines for patients with DS who underwent lumbar fusion surgery through a comparison of functional outcomes preoperatively and at six months following surgery. Patient demographics, clinical features, and PROMs were collected. This study was exempted from patient consent and was approved by an institutional review board (IRB).

### 2.2. Participants, Variables, and Data Measurement

**Figure 1:**
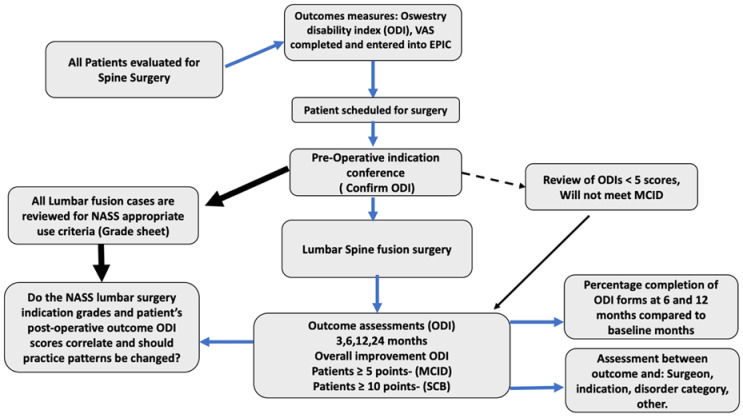
Flowchart describing the patient selection process for surgery.

All cases who underwent elective lumbar fusion surgery for DS from March 2018 until August 2019 were reviewed. All cases were carefully evaluated for compliance with EBM guidelines by a group of neurosurgeons led by a senior spine neurosurgeon. Each lumbar fusion case was categorized as either EBM concordant or EBM discordant. Films were reviewed and cases categorized by EBM indication. If no EBM category were appropriate, the patient was labeled EBM discordant or “not indicated.” The patients were followed, and if ODI improved greater than 5 points were achieved, they achieved the MCID. Surgical candidates were chosen by meticulously reviewing the NASS criteria indications and following objective patient-reported outcome measures (PROMs) (Figure 1). Inclusion criteria included patients over 18 years of age undergoing a lumbar operative fusion in an elective manner. All patients with acute trauma or undergoing fusion in an emergent manner were excluded from this study. A total of 146 patients met the inclusion criteria. Of these, 125 were considered EBM concordant, while 21 were considered EBM discordant. The ODI was chosen as the PROM and completed at each neurosurgical spine evaluation.

Preoperative and six-month postoperative ODI scores were collected. Clinical outcomes were divided into: substantial clinical benefit defined as ODI change greater or equal to 10 points (SCB ΔODI ≥ 10 points), substantial clinical benefit (SCB) thresholds for ODI defined as a net improvement of 18.8 points, and a 36.8% improvement or a final raw score of 31.3 points [7]. Minimal clinically importance difference, which represents the critical point of change from baseline defined as ODI change greater than or equal to 5 points (MCID ΔODI ≥ 5) was used. This cutoff for MCID was chosen based on an anchor-based analysis by Monticone et al. that reported a 4.8-point improvement to be an optimal cutoff for this dichotomous outcome (sensitivity 76% and specificity 63%) [8]. No MCID was defined for patients whose ODI improved but did not reach 5 points (ΔODI 1–4 points) and a group that showed no change or worsening ODI. Student’s *t*-test was used to compare the mean ODI scores. All ODI scores are displayed as raw scores (0–50 points) and not as percentage disability (0–100).

### 2.3. Statistical Analysis

Data were collected and transferred for analysis to SPSS 26.0 for Windows (Chicago, IL, USA) and Microsoft Excel 2016. Descriptive statistics were used to summarize the distribution of the data. Preoperative and postoperative mean ODI scores were compared using paired and unpaired two-tailed Student’s *t*-test. Fisher’s exact test was performed to study the correlation between the indications of spine fusion following EBM guidelines and MCID in the ODI score at six-month follow-up. Furthermore, Fisher’s exact test was performed to study the correlation between gender, diabetes mellitus, osteoporosis, smoking, back pain, leg pain, complications, spinal cord stimulator (SCS) use, revision surgery, and surgical procedure. Chi-squared was used to demonstrate the relation between the type of procedure and indication status. American Society of Anesthesiology (ASA) score and body mass index (BMI) were studied using a Mann–Whitney U test. Normality was assessed using visual inspection of variable histograms and Q-Q plots and verified using the Kolmogorov–Smirnov normality test. A nonparametric test was used for any variable showing abnormal distribution. Logistic regression was performed to determine the associations between EBM criteria compliance (independent variable) and functional outcomes (dependent variable). Statistical evaluations were two-sided, and a *p*-value < 0.05 was set for statistical significance.

## 3. Results

Overall, 85.61% (125/146) of the lumbar fusion patients met the EBM fusion criteria and are referred to as EBM concordant, while 14.38% (21/146) did not meet the criteria and are referred to as EBM discordant. The statistical test was computed at a 95% level of confidence.

Analysis of 125 DS (EBM concordant) patients was performed comparing preoperative and postoperative (six months) ODI scores. In this series, females represented the majority of the population studied, accounting for 64% (92/146) of the population, with a mean age of 64.91 ± 10.1 years. Males accounted for 36.0% (54/146) of the group with a mean age of 67.17 ± 9.75 years. (*p* = 0.9 and 0.14 for age and gender, respectively). The mean BMI identified was 30.36 ± 5.70 (*p* = 0.43), and 37.19% (47/146) of patients presented with a history of DM (*p*= 0.13). Osteoporosis was seen in 59.6% (87/146) of the population (*p* = 0.63). Mean ASA score was 2.6 ± 0.5 (*p* = 0.47), and 12.3% had a smoking history (*p* = 0.29) (Table 1).

The most common presenting clinical condition was low-back pain, which was present in 98.6% (144/146) of the patients. Furthermore, lower-extremity radicular pain was present in 92.46% (135/146) of patients. The mean baseline ODI score for DS patients was 23.68 ± 8.8. These patients had a significant clinical improvement based on ODI scores at six months compared to their baseline scores. Specifically, the ODI improved 8 points from 23.68 ± 8.8 to 15.68 ± 8.9 (*p* < 0.0001) in the EBM concordant group vs. 2.14 points from 21.1 ± 9.63 to 19 ± 10.28 (*p* = 0.23) in the EBM discordant group. Fisher’s exact test showed statistical significance in comparing both EBM concordant and EBM discordant fusion groups (*p* = 0.02). (Table 2).

### 3.1. Surgical Procedures: Oswestry Disability Index Subanalysis

In the population concordant with the EBM criteria, the most common surgical approach used was TLIF in 75% (94/125), followed by extreme lateral interbody fusion (XLIF^®®^) in 8.8% (11/125). In EBM discordant cases, the most common surgical approach used was TLIF in 57.14% (12/21). A total of 14% (11/21) underwent posterior lumbar interbody fusion (PLIF) (*p* = 0.003). The distribution of the rest of the procedures is thoroughly described in Table 1.

### 3.2. Oswestry Disability Index Subanalysis

A subanalysis was conducted between the 125 patients who met the EBM criteria (EBM concordant) and those who did not (EBM discordant). Overall, 81.6% (102/125) of the EBM concordant patients showed improvement in their ODI score at six months. SCB (ΔODI >10 points) was obtained in 42% (52/125) of the patients, while 64.8% (81/125) showed MCID (ΔODI ≥ 5 points), and 16.8% (21/125) showed improvement but did not meet the MCID (ΔODI 1–4 points). Overall, 18.4% (23/125) of patients had ODI scores that did not improve. Of these, 4.8% (6/125) were unchanged, and 13.6% (17/125) declined in their ODI score (Table 2).

**Figure 2:**
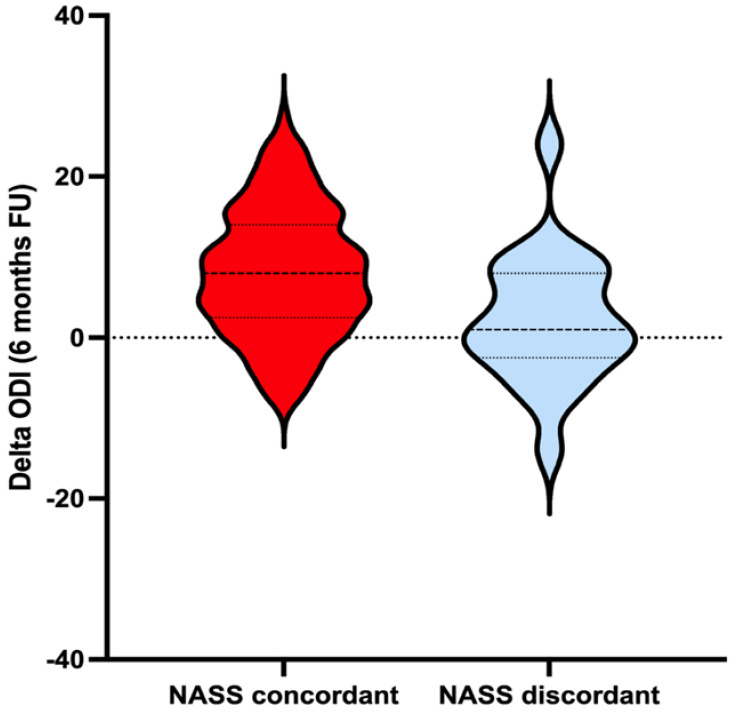
Violin plot for the distribution of change in ODI, stratified by EBM concordant degenerative spondylolisthesis fusion surgery and EBM discordant population vs. postoperative ODI scores after 6 months’ follow-up. Violin plot upper/lower bounds are the 25% and 75% limits (interquartile range) and represented by dotted lines. Solid lines represent mean delta ODI. The width of the violin indicates the distribution of the change in the patient’s ODI after six months of follow-up.

A total of 51% (12/21) of the EBM discordant patients showed improvement in their ODIs at six months. SCB was reached in 14% (3/21) of the patients, while 38.1% (8/21) showed MCID and 19% (4/21) showed improvement but did not meet the MCID (ΔODI 1–4 points), while 42.8% (9/21) of patients had ODI scores that did not improve. Of these, 4.76% (1/21) were unchanged, and 38% (8/21) declined (Table 2). Most of the EBM concordant group reached an MCID compared to the EBM discordant group, achieving statistical significance for this finding (*p* = 0.002, Figure 2—violin plot). Compliance with EBM guidelines was associated with an odds ratio (OR) of 2.93 for achieving MCID ([CI]: 1.128–7.58, *p* = 0.027) [9].

## 4. Discussion

Degenerative spondylolisthesis is a condition that commonly affects more women than men [1,2]. Over 64% of the population was female (92/146), with a mean age of 64.91 ± 10.1 years [10]. Ghogawala et al. suggested that decompressing and fusing patients with DS may be more beneficial than just decompression alone [11]. This study further demonstrates that patients meeting EBM criteria may benefit more from lumbar fusion than those who do not meet the criteria [5,11,12,13,14,15,16].

The EBM concordant population showed a significant improvement in ODI scores at six-month follow-up. This is in contrast to the EBM discordant group, which did not show a statistically significant improvement in ODI scores at six months following surgery (*p* = 0.23, Table 2). The baseline ODI scores obtained in this study were similar to those found in a study conducted by Asher et al., where they observed an improvement of 6.1 points between baseline and one-year ODI scores in patients undergoing elective lumbar fusion for DS [12,17]. In this study, patients received follow-up after six months, and an improvement of 8 points in ODI was obtained. In a comparative analysis reported by Herkowitz et al., 25 patients with DS were treated with decompression and fusion. Of these, 44% reported excellent, 52% good, 4% fair, and 0 poor results. Patients with an arthrodesis concomitantly with decompression had a statistically significant improvement in outcome by Fisher’s exact test (ρ = 0.0001) [18]. Although this study showed similar results, we have identified that the type of surgery indicated to treat this condition remains unclear these days. In a meta-analysis published by Ahmed et al., decompression with fusion was a 3.5 times better surgical technique than decompression alone for spinal stenosis in terms of Oswestry Disability Index and visual analogue pain scale for back and leg pain [19]. This, however, contrasts with the current NASS guidelines, which recommend decompression only for patients with lumbar stenosis, predominantly leg pain if there is no evidence of spondylolisthesis or instability.

These results were similar to those found in the EBM concordant population, where 81% (102/125) of patients were found to have improved ODIs at six months, compared to only 51% (12/21) of patients observed to have ODI improvement in the EBM discordant group (*p* < 0.04, Table 2) [20]. This finding further supports that patients undergoing lumbar fusion in agreement with EBM criteria may have better functional outcomes [9]. In this series, SCB was reached in 42% of the patients and 64.8% showed MCID. The EBM discordant group was a small percentage of the total patient lumbar fusion population, and 14.38% (21/146) of patients had a mean ODI improvement of only 2.14 points compared to 8 points for the EBM concordant patients (*p* = 0.002) (Table 2 and Figure 2). Of the EBM discordant group, 38.1% (8/21) met the MCID (ΔODI ≥ 5) for ODI improvement at six months versus 64.2% (81/125) in the EBM concordant group (*p* = 0.02) (Table 2).

In patients who received care in concordance and discordance with evidence-based medicine (EBM), failed back surgery syndrome (FBSS), pseudoarthrosis, and adjacent level degeneration were found to be the most prevalent complications [21]. This study determined that 17.6% (22/125) of patients who received care in accordance with EBM underwent revision surgery. Notably, it was determined that this rate was higher among patients who received care that was discordant with EBM, with 23.8% (5/21) requiring revision surgery. This outcome suggests that adherence to EBM guidelines may be associated with a lower rate of revision surgery. However, it is essential to consider that other factors, such as patient characteristics, surgical technique, and follow-up care, may also play a role in the incidence of revision surgery (Table 2).

Finally, at a quaternary care facility, numerous patients who have exhausted all non-operative strategies are treated. While guidelines are beneficial, they do not always provide direct care for refractory patients. Surgery is provided as an option, and several patients opt for surgical care. In addition, surgeons may only follow guidelines for some care, instead relying on their professional judgment.

## 5. Limitations

There are several limitations to this study. This was a retrospective analysis, and it will be worth seeing these analyses performed on multiple health centers. Further studies should include a more significant number of patients with discordant EBMs. Also, the degree of listhesis, severity of stenosis, and symptoms should be another group of factors that might be related to the clinical outcomes of the patients but were not directly studied in this series.

## 6. Conclusions

Patients whose lumbar fusions meet EBM criteria had better self-reported outcomes at six months than those who did not meet the criteria. Based on the data presented in this study, surgeons following evidence-based criteria for lumbar fusion were able to achieve better functional outcomes.

## Figures and Tables

**Table 1:** Demographic analysis of patients based on their concordance with the evidence-based medicine (EBM) guidelines.

Variables	Overall (n = 146)	EBM Concordant (n = 125)	EBM Discordant (n = 21)	*p* Values (χ2 Test, Fisher’s Exact Test, Paired 2-Tailed Student’s *t*-Test and Unpaired 2-Tailed Student’s *t*-Test, Mann–Whitney U Test
Age	65.75 (64.11–67.4)	65.92 (64.24–67.60)	64.71 (58.8–70.61)	0.9
Gender Male Female	36% (54/146) 64% (92/146)	34.4% (43/125) 65.6% (82/125)	52.3% (11/21) 47.7% (10/21)	0.14
BMI	30.36 (29.4–31.3)	30.52 (29.503154)	29.4 (26.93–31.87)	0.43
Diabetes Y N	32.19% (47/146) 67.8% (99/146)	29.6% (37/125) 70.4% (88/125)	47.6% (10/21) 52.3% (11/21)	0.13
Osteoporosis Y N	59.6% (87/146) 40.4% (59/146)	41.6% (52/125) 58.4% (73/125)	33.3% (7/21) 66.7% (14/21)	0.63
ASA score	2.6 (2.5–2.7)	2.6 (2.5–2.7)	2.52 (2.29–2.75)	0.47
Smoking Y N	12.3% (18/146) 87.6% (128/146)	11.2% (14/125) 88.8% (111/125)	19% (4/21) 81% (17/21)	0.29
Back PainY N	98.6% (144/146) 1.4% (2/146)	99.2% (124/125) 0.8% (1/125)	95.23% (20/21) 4.7% (1/21)	0.26
Leg pain Y N	92.46% (135/146) 7.54% (11/146)	92% (115/125) 8% (10/125)	95.23% (20/21) 4.7% (1/21)	0.9
Complications Y N	13.7% (20/146) 86.3% (126/146)	12% (15/125) 88% (110/125)	23.8% (5/21) 76.2% (16/21)	0.72
Spinal Cord Stimulator Y N	11% (16/146) 89% (110/146)	10.4% (13/125) 89.6% (112/125)	14.2% (3/21) 85.7% (18/21)	0.70
Revision Surgery Y N	18.4% (27/146) 81.6% (119/146)	17.6% (22/125) 82.4% (103/125)	23.8% (5/21) 76.1% (16/21)	0.5446
Type of procedure TLIF XLIF PLF AP ALIF PLIF PAP Percutaneous	73% (106/146) 8.9% (13/146) 7.4% (11/146) 4.1% (6/146) 3.4% (5/146) 2% (3/146) 0.6% (1/146) 0.6% (1/146)	75.2% (94/125) 8.8% (11/125) 8% (10/125) 3.2% (4/125) 3.2% (4/125) 0% (0/125) 0.8% (1/125) 0.8% (1/125)	57.14% (12/21) 9.52% (2/21) 4.76% (1/21) 9.52% (2/21) 4.76% (1/21) 14.28% (3/21) 0% (0/21) 0% (0/21)	0.003

**Table 2:** Distribution of change in ODI, stratified by EBM concordant degenerative spondylolisthesis fusion surgery and EBM discordant population vs. postoperative ODI scores after 6 months’ follow-up.

Variables	Overall (n = 146)	EBM Concordant (n = 125)	EBM Discordant (n = 21)	*p* Values (χ2 Test, Fisher’s Exact Test, Paired 2-Tailed Student’s *t*-Test and Unpaired 2-Tailed Student’s Test, Mann–Whitney U Test)
Preoperative ODI	23.1 (21.84–24.77)	23.68 (22.12–25.24)	21.1 (16.7–25.5)	0.19
Postoperative ODI	16.2 (14.7–17.7)	15.68 (14.1–17.3)	19 (14.27–23.63)	0.04
Delta ODI	7.2 (5.8–8.4)	8 (6.6–9.39)	2.14 (−1.54–5.28)	0.002
SCB	37.7% (55/146)	41.6% (52/125)	14.2% (3/21)	0.01
MCID	61% (89/146)	64.8% (81/125)	38% (8/21)	0.02
No MCID	17.1% (25/146)	16.8% (21/125)	19% (4/21)	0.78
Declined	4.79% (7/146)	13.6% (17/125)	4.76% (1/21)	-
No Change	17.1% (25/146)	4.8% (6/125)	38% (8/21)	-
OR		2.93 ([CI]: 1.128–7.58, *p* = 0.027)		

## Data Availability

Data is unavailable due to privacy or ethical restrictions.
